# Exploring the role of Prx II in mitigating endoplasmic reticulum stress and mitochondrial dysfunction in neurodegeneration

**DOI:** 10.1186/s12964-024-01613-x

**Published:** 2024-04-18

**Authors:** Mei-Hua Jin, Lin Feng, Hong-Yi Xiang, Hu-Nan Sun, Ying-Hao Han, Taeho Kwon

**Affiliations:** 1 College of Life Science & Biotechnology Technology, Heilongjiang Bayi Agricultural University, 163319 Daqing, China; 2 Primate Resources Center, Korea Research Institute of Bioscience and Biotechnology (KRIBB), 351-33 Neongme-gil, Ibam-myeon, 56216 Jeongeup-si, Jeonbuk, Republic of Korea; 3 Department of Applied Biological Engineering, KRIBB School of Biotechnology, National University of Science and Technology (UST), 34113 Daejeon, Republic of Korea

**Keywords:** Neurodegenerative diseases, Peroxiredoxin II, Reactive oxygen species, Endoplasmic reticulum-mitochondrial interactions, Mitochondrial damage

## Abstract

**Background:**

Neurodegenerative diseases are increasingly recognized for their association with oxidative stress, which leads to progressive dysfunction and loss of neurons, manifesting in cognitive and motor impairments. This study aimed to elucidate the neuroprotective role of peroxiredoxin II (Prx II) in counteracting oxidative stress-induced mitochondrial damage, a key pathological feature of neurodegeneration.

**Methods:**

We investigated the impact of Prx II deficiency on endoplasmic reticulum stress and mitochondrial dysfunction using HT22 cell models with knocked down and overexpressed Prx II. We observed alcohol-treated HT22 cells using transmission electron microscopy and monitored changes in the length of mitochondria-associated endoplasmic reticulum membranes and their contact with endoplasmic reticulum mitochondria contact sites (EMCSs). Additionally, RNA sequencing and bioinformatic analysis were conducted to identify the role of Prx II in regulating mitochondrial transport and the formation of EMCSs.

**Results:**

Our results indicated that Prx II preserves mitochondrial integrity by facilitating the formation of EMCSs, which are essential for maintaining mitochondrial Ca^2+^ homeostasis and preventing mitochondria-dependent apoptosis. Further, we identified a novel regulatory axis involving Prx II, the transcription factor ATF3, and miR-181b-5p, which collectively modulate the expression of Armcx3, a protein implicated in mitochondrial transport. Our findings underscore the significance of Prx II in protecting neuronal cells from alcohol-induced oxidative damage and suggest that modulating the Prx II-ATF3-miR-181b-5p pathway may offer a promising therapeutic strategy against neurodegenerative diseases.

**Conclusions:**

This study not only expands our understanding of the cytoprotective mechanisms of Prx II but also offers necessary data for developing targeted interventions to bolster mitochondrial resilience in neurodegenerative conditions.

**Supplementary Information:**

The online version contains supplementary material available at 10.1186/s12964-024-01613-x.

## Introduction

Neurodegenerative diseases, characterized by the progressive loss of neurons within the central nervous system, represent a significant medical challenge [1]. These disorders are diverse and complex, and their pathogenesis involves genetic, environmental, and molecular factors. However, oxidative stress is a unifying factor, and is consistently implicated in the development and progression of neurodegenerative conditions [2–4]. Oxidative tissue damage is increasingly being recognized as a critical player in the pathogenesis of neurodegenerative diseases.

Proteins of the peroxiredoxin family, particularly peroxiredoxin II (Prx II), play a crucial role in cellular defense against oxidative stress [5–7]. Prx II is localized in the neurons of the gray matter, including the hippocampus, cerebral cortex, and thalamus [8]. Studies, including those examining alcohol-induced mitochondrial damage in hippocampal neuronal cells HT22, have highlighted the protective role of Prx II in regulating reactive oxygen species (ROS) [9]. These collective insights suggest that Prx II is a potential therapeutic target for neurodegenerative diseases that are linked to oxidative stress and mitochondrial damage.

This study examined the neuroprotective mechanisms of Prx II, focusing on its ability to counteract mitochondrial damage caused by oxidative stress. Given the profound neurotoxic effects of alcohol, we selected it as the neurodegenerative factor examined in this study. The association between chronic alcohol intake and the decline in cognitive function, along with the onset of neurodegenerative processes, positions alcohol as an apt model for probing the molecular basis of these debilitating conditions. Alcohol promotes both oxidative stress and endoplasmic reticulum (ER) stress (ERS), functions that are integral to the development of neurodegenerative diseases. We utilized transmission electron microscopy, on HT22 cells treated with alcohol, to document significant alterations in the length of the mitochondria-associated ER membranes and the frequency of the contact sites between these organelles, especially in cells where Prx II expression had been diminished. Our findings suggest that Prx II may protect against mitochondrial damage by modulating ER-mitochondrial contact sites (EMCSs), which are critical for mitochondrial Ca^2+^ homeostasis and overall mitochondrial function [10–12]. Further investigation into the regulatory role of Prx II in EMCSs revealed potential mechanisms by which it may influence mitochondrial Ca^2+^ levels, function, and cell survival. These insights contributed to our understanding of the role of Prx II in neuroprotection, and highlighted its potential as a therapeutic target for treating neurodegenerative diseases caused by oxidative stress.

This study investigated the protective effects of Prx II against ERS-induced mitochondrial dysfunction, which are key contributors to neurodegenerative processes, and aimed to clarify the underlying molecular mechanisms.

## Materials and methods

### Experimental design

Using the HT22 cell line, we genetically altered Prx II levels and exposed the cells to 400 mM alcohol for 1 h to simulate neurodegenerative conditions. Post-treatment assessments included mitochondrial function, ERS markers, and the analysis of protein and miRNA expression in the Prx II pathway to determine its role in cellular defense against alcohol-induced stress.

### Cell culture

HT22 cells were obtained from the ATCC (Manassas, VA, USA). The cells were cultured in high-glucose DMEM supplemented with 10% fetal bovine serum, penicillin (100 U/mL), and streptomycin (100 µg/mL) at 37 ℃, 5% CO_2_, and 95% air. The cells were further divided into different groups according to the experiment: a blank control group (mock HT22), a lentiviral knockdown group (shPrx II HT22), and a lentiviral overexpression group (wtPrx II HT22) (Gima Genetics Ltd., Suzhou, China).

### Confocal microscope analysis

HT22 cells were seeded in 15 mm confocal dishes and divided into control (0 mM alcohol) and alcohol-treated (400 mM alcohol) groups to assess their response to oxidative stress. Cell counting was performed to ensure that the cells were inoculated at a density of 2 × 10^5^ cells per dish, in 2 mL medium. After 24 h of culture, the medium was changed with one containing 1% fetal bovine serum, for cell starvation for approximately 2 h. After 2 h, the cells were treated with alcohol for 1 h in the corresponding medium. The cells were fixed with 4% paraformaldehyde for 10 min, and after fixation, Hoechst and Cell Navigator™ Mitochondrion Staining Kit dyes were added; the cells were further incubated for 20 min at 37 ℃, protected from light, and images were captured using a Leica laser confocal scanning microscope.

### Transmission electron microscopy analysis

HT22 cells were exposed to alcohol and incubated overnight at 4 ℃ in a fixative solution consisting of 4% paraformaldehyde and 1% glutaraldehyde. The cells were rinsed with phosphate-buffered saline and fixed with 1% osmium tetroxide. After fixation, ethanolic dehydration, Epon embedding, and polymerization were performed. Next, the cells were cut using an ultramicrotome, stained with 1% uranyl acetate and lead citrate, and examined under a transmission electron microscope (JEOL JEM-2100Plus, Nippon Electronics, Tokyo, Japan).

### Mitochondrial Ca^2+^ detection

To detect Ca^2+^ in mitochondria, a specific dye for mitochondrial Ca^2+^ uptake, Rhod-2, AM (R1245MP, Invitrogen, Thermo Fisher Scientific Inc., Waltham, MA) [13], was used. Briefly, the stock solution of Rhod-2, AM was dissolved in DMSO and incubated with Rhod-2 at a concentration of 0.25 µM and Hoechst at a concentration of 1 ug/mL, for 30 min at 37 ℃, in the dark. The cells were washed thrice to remove excess or non-specific probes loaded into the mitochondria, and the fluorescence intensity was analyzed using flow cytometry.

### RNA-sequencing

RNA was isolated from HT22 cells after alcohol treatment, using TRIzol, following the manufacturer’s instructions. The RNA samples were sent to Paisano Shanghai for further analysis. They determined RNA concentration, quality, and integrity using a Nanodrop spectrophotometer, constructed sequencing libraries, and finally sequenced them using the NovaSeq 6000 platform (Illumina) to obtain differential genes and performed gene ontology (GO) analysis of the differentially expressed genes.

### Bioinformatics analysis

To predict the miRNAs that can regulate Armcx3, we input Armcx3 into the miRWalk [14], miRDB [15], and StarBase [16] databases, selected the mice intersecting the miRNAs predicted by the miRWalk, miRDB, and StarBase databases, and plotted Venn diagrams to obtain the common target miRNAs.

To predict the transcription factors regulating miR-181b-5p, we first obtained the miR-181b-1 and miR-181b-2 promoter sequences of the miR-181b-5p precursor from the NCBI database, copied them to the JASPAR database [17] with a default threshold score of 85.0, and obtained two potential CDS regions of the transcription factor ATF3 miR-181b-1 promoter binding sites and seven potential miR-181b-2 promoter binding sites.

### Immunofluorescence staining

HT22 was inoculated in 24-well plates at a density of 8,000 cells per well and treated with 400 mM alcohol for 1 h (37 ℃, 5% CO_2_). The mitochondrial membrane potential was detected using the fluorescent probe JC-1, following the manufacturer’s protocol. Lastly, fluorescence microscopy imaging was performed using Leica Microsystems (Wetzlar, Germany).

### Quantitative real-time PCR

Total RNA was extracted from the cells using TRIzol (Sangon Biotech, Shanghai, China). Next, the total RNA was reverse transcribed to cDNA using the Nova UScript II All in One First-Strand cDNA Synthesis SuperMix kit (Invitrogen, Carlsbad, CA, USA). We measured mRNA expression levels using the 2 × InNova Taq SYBR Green qPCR Mix kit. β-actin was used as an endogenous control. The primer sequences were synthesized and designed by Sangon Biotech (Shanghai, China) and are listed in Table 1. The first strand of miRNA was used, as per the manufacturer’s instructions, and a cDNA synthesis kit (tailing method) was used to reverse-transcribe the total RNA into cDNA. The miRNA expression levels were detected using an miRNA fluorescence quantitative PCR kit (dye method). Primer sequences were synthesized and designed by Sangon Biotech (Shanghai, China) and are listed in Table 1. Data are expressed as relative Ct values. The 2-DDCt method was used to calculate the relative expression levels.

**Table 1:** List of differentially expressed genes

Gene ID	Gene name	Log_2_FC	Regulate
ENSMUSG00000025733	Rhot2	+ 0.469589609961882	up
ENSMUSG00000020741	Cluh	+ 0.293542693911011	up
ENSMUSG00000049047	Armcx3	-0.269899972721329	down

### Western blotting

For each sample, total protein was separated using SDS-PAGE (12% gel) and electrotransferred onto nitrocellulose membranes (Millipore, Bedford, MA, USA). The membranes were washed five times with Tris-buffered saline. The following primary antibodies were used: anti-Bax (Santa Cruz Biotechnology, Santa Cruz, CA, USA), anti-Bcl2 (Santa Cruz Biotechnology, Santa Cruz, CA, USA), anti-caspase-3 (Santa Cruz Biotechnology, Santa Cruz, CA, USA), anti-ATF3 (Sangon Biotech, Shanghai, China), anti-PERK (Sangon Biotech, Shanghai, China), anti-p-PERK (Sangon Biotech, Shanghai, China), anti- Armcx3 (Sangon Biotech, Shanghai, China), and anti-β-actin (Santa Cruz Biotechnology, Santa Cruz, CA, USA). Goat anti-mouse (ZSGB-BIO, Beijing, China) and anti-rabbit (ZSGB-BIO) secondary antibodies were used. The blots were photographed using Alpha View Software (AlphaView, USA) and analyzed using ImageJ software.

### Statistical analysis

All data were analyzed using the GraphPad Prism 8 software and are presented as the mean ± SEM. We employed t-tests and two-way analyses of variance to evaluate statistical significance, which was set at *P* < 0.05.

## Results

### Prx II attenuates alcohol-induced mitochondrial dysfunction via EMCSs modulation

**Fig. 1:**
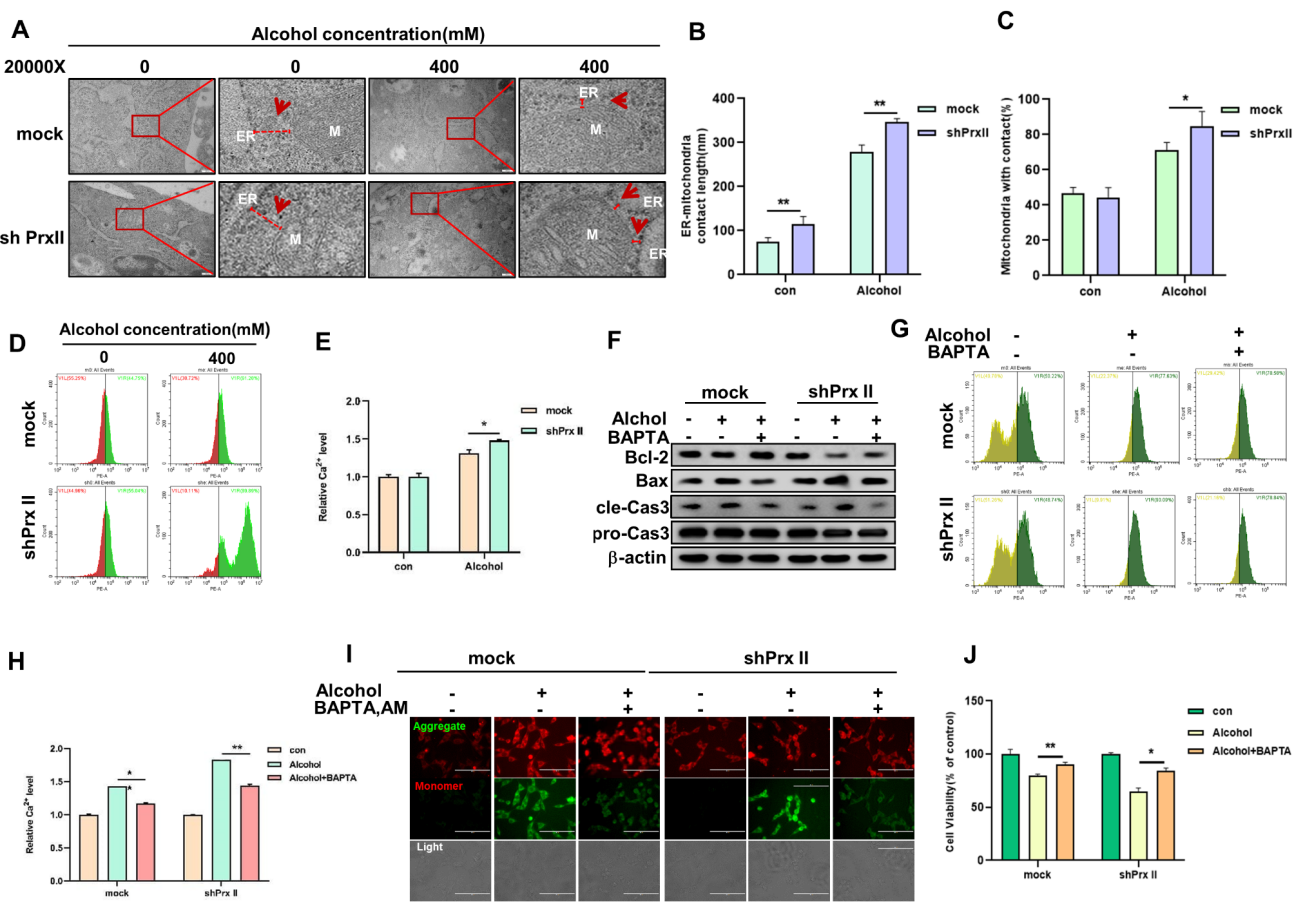
Prx II regulation of EMCS formation in HT22 inhibits alcohol-induced decrease in mitochondrial membrane potential. (**A**) Analysis of EMCS formation using transmission electron microscopy, with the ER labeled as ER and mitochondria labeled as M. The distance between the ER and mitochondria is marked with red dashed lines. (**B**) Quantification of the length of EMCS. (**C**) The percentage of mitochondria in contact with ER. These images are representative of three independent experiments, each with *n* = 3 technical replicates. (**D**) Flow cytometry detection of intra-mitochondrial Ca^2+^ levels; (**E**) Quantitative analysis of the data in panel “d.” (**F**) Western blotting detection of mitochondria-dependent apoptosis-related protein expression levels after BAPTA pretreatment. (**G**) Cytometry detection of intra-mitochondrial Ca^2+^ levels after BAPTA pretreatment. (**H**) Quantitative analysis of the data in panel “g.” (**I**) Fluorographic detection of mitochondrial membrane potential after BAPTA pretreatment. (**J**) MTT assay detection of BAPTA pretreatment after alcohol-induced HT22 cell death

This study revealed that Prx II plays a pivotal role in preserving mitochondrial membrane potential in HT22 cells challenged with alcohol [9]. This is accomplished by modulating the formation of EMCSs, a process crucial for maintaining intra-mitochondrial Ca^2+^ homeostasis and mitochondrial function [10, 11]. Transmission electron microscopy analysis demonstrated a significant increase in the length of mitochondria-associated ER membranes and the percentage of mitochondria in contact with the ER in Prx II-deficient cells post-alcohol exposure (Fig. 1A–C). Additionally, intra-mitochondrial Ca^2+^ levels were elevated in these cells (Fig. 1D and E). However, treatment with the Ca^2+^ chelator BAPTA ameliorated these effects, restoring mitochondria-dependent apoptosis, mitochondrial membrane potential, and cell viability (Fig. 1F-J and Fig. S1). These findings suggest that Prx II maintains mitochondrial membrane potential by modulating EMCSs and intra-mitochondrial Ca^2+^ levels.

### Prx II modulates mitochondrial subcellular localization through Armcx3

**Fig. 2:**
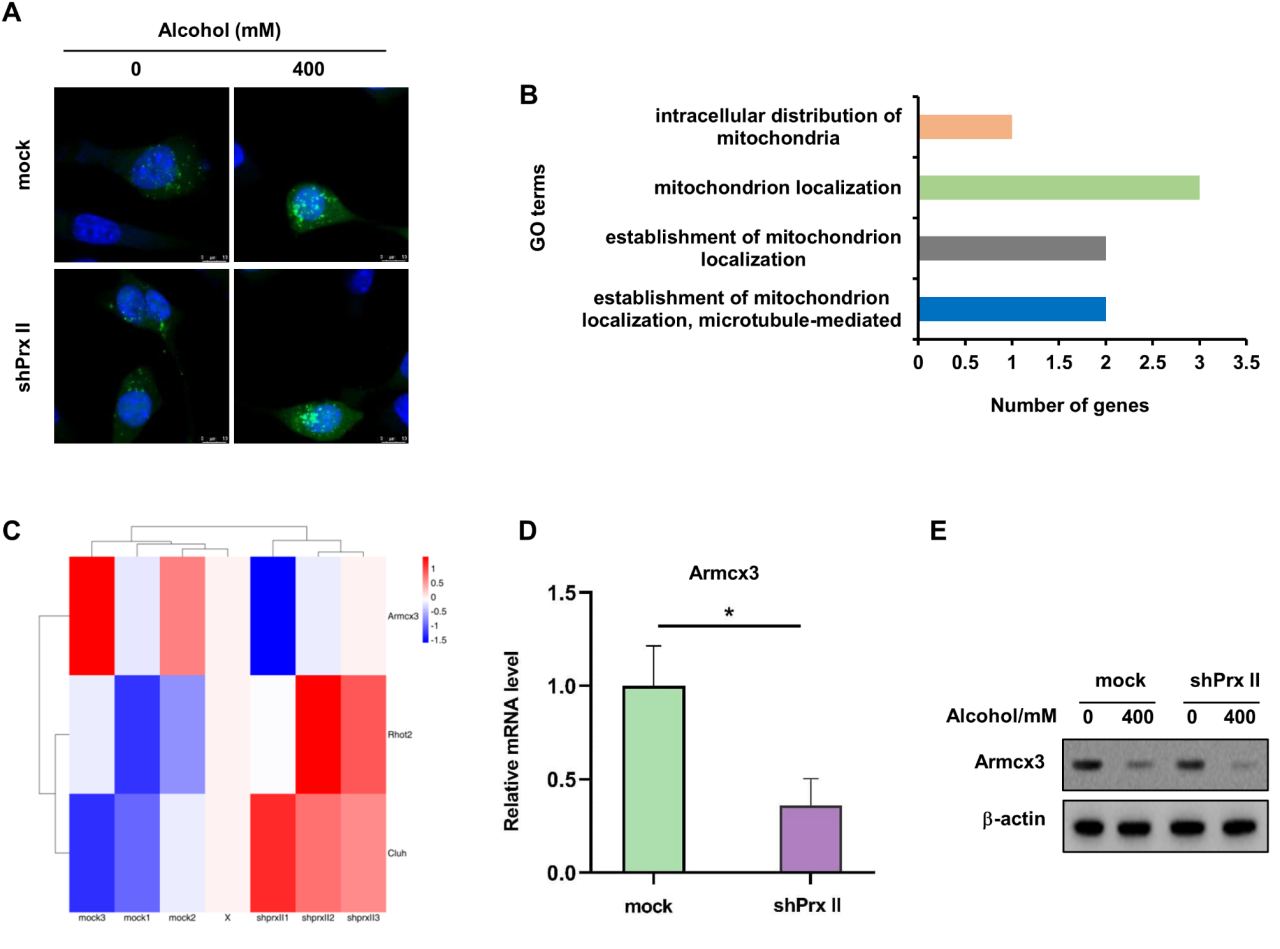
Prx II regulates Armcx3 mitochondrial subcellular localization to regulate EMCS formation. (**A**) Confocal microscopy was used to detect mitochondrial subcellular localization. (**B**) RNA-seq analysis of GO of mock HT22 and shPrx II HT22 cells involved in mitochondrial subcellular localization after alcohol treatment. (**C**) RNA-seq analysis of differentially expressed genes involved in the mitochondrial subcellular localization of mock HT22 and shPrx II HT22 cells after alcohol treatment and plotting heat map; red and blue indicate upregulated and downregulated genes, respectively. (**D**) Armcx3 mRNA expression levels were verified using qRT-PCR. (**E**) Western blotting was used to detect Armcx3 protein expression levels. Each analysis was performed in triplicate

The localization of mitochondria within cells affects EMCS formation [12]. We investigated the impact of Prx II on mitochondrial subcellular localization, and found that mitochondria aggregate around the nucleus in shPrx II HT22 cells, following alcohol treatment, indicating a disruption in mitochondrial distribution (Fig. 2A). RNA sequencing identified changes in gene expression related to mitochondrial localization, with a notable downregulation of Armcx3 [18] in shPrx II HT22 cells (Fig. 2B–E; Figs. S2 and S3), as evidenced by the data presented in Table 2. This downregulation was confirmed at both the protein and RNA levels. These results indicate that Prx II modulates mitochondrial aggregation and EMCS formation via Armcx3.

**Table 2:** miRNA genes

	Forward primer(5’-3’)		Forward primer(5’-3’)
miR-181b-5p	AACATTCATTGCTGTCGGTGG	miR-494-3p	AAGAAGATGAAACATACACGGG
miR-181d-5p	CACTGCCTAACATTCATTGTTG	miR-181c-5p	AACACGCAACATTCAACCTG
miR-181a-5p	ACACCAAACATTCAACGCTG	miR-145a-5p	AAGCTTAGTCCAGTTTTCCCA
miR-23a-5p	AATAGTGGGGTTCCTGGGG	miR-5129-5p	AAGCTTAATGTGGGGGCATT
miR-342-3p	AACTTCTCTCACACAGAAATCG	miR-8106	AAGAGCGTTGACTCTGTACAT
miR-3090-5p	GGTGGGGCCTGAGATC	miR-7093-5p	AAGCACCAGGATGACAGAAG
miR-1249-5p	AAGAATTAGGAGGGAGGGGAT	miR-3072-3p	CCCTCCAGGAAGCCTTCT
miR-6912-3p	AAGAAGACTACCTGAGGGTCT	miR-8104	AATGCTTCAGGATGAGGTGG
miR-7075-5p	GGGAGGAGGACATGGTTTT	miR-201-5p	AATCCGGATACTCAGTAAGGC
miR-669c-3p	AACAAGTACACACACACACAC		

### Prx II mediates ERS-dependent mitochondrial protection via Armcx3

**Fig. 3:**
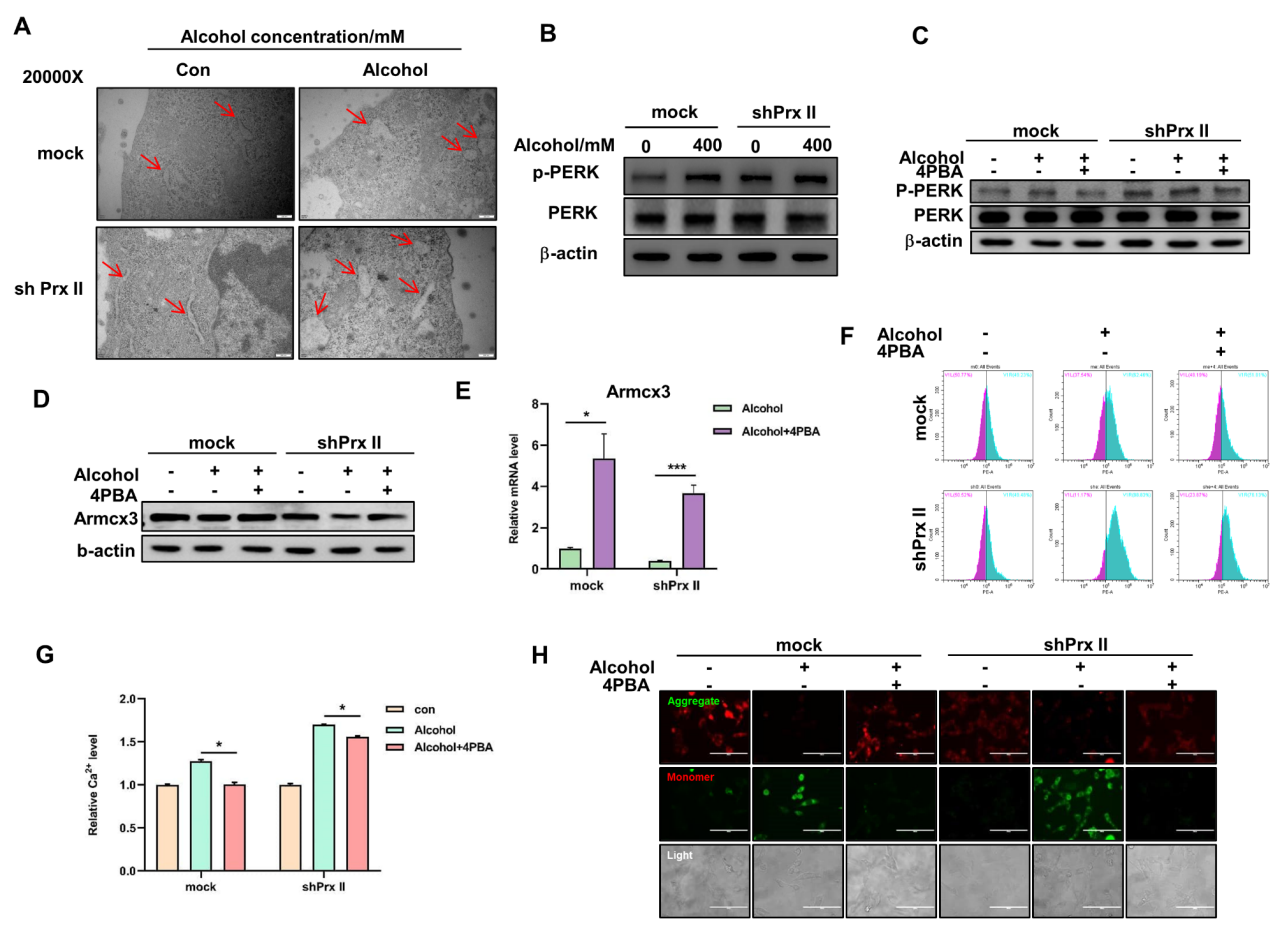
Prx II regulates Armcx3 expression via ERS. (**A**) Detection of ERS within HT22 cells after alcohol treatment using projection electron microscopy. (**B**) Western blotting was used to detect PERK signaling pathway protein expression levels. (**C**) Detection of PERK signaling pathway after 4PBA pretreatment using western blotting. (**D**) Armcx3 protein expression level after 4PBA pretreatment, assessed using western blotting. (**E**) Armcx3 expression level after 4PBA pretreatment, assessed using qRT-PCR. (**F**) Ca^2+^ level in the mitochondria after 4PBA pretreatment, assessed using flow cytometry. (**G**) Quantitative analysis of the data in panel “f.” (**H**) Mitochondrial membrane potential after 4PBA pretreatment, assessed using fluorography. Each analysis was performed in triplicate

Alcohol-induced neuronal cell death is often mediated by ERS [19]. We further explored the link between Prx II and protection against alcohol-induced mitochondrial dysfunction mediated by ERS. Our results showed that the ER was significantly swollen and the PERK signaling pathway was significantly activated in Prx II-deficient cells post-alcohol treatment (Fig. 3A, B and S4A). The application of the ERS inhibitor 4PBA led to the suppression of PERK signaling, upregulation of Armcx3, and the normalization of intra-mitochondrial Ca^2+^ levels and mitochondrial membrane potential (Fig. 3C–H and S4B–D). These findings indicate that Prx II can modulate ERS to prevent alcohol-induced mitochondrial dysfunction, with Armcx3 as the downstream target.

### Prx II suppresses Armcx3 expression via miR-181b-5p and ATF3

**Fig. 4:**
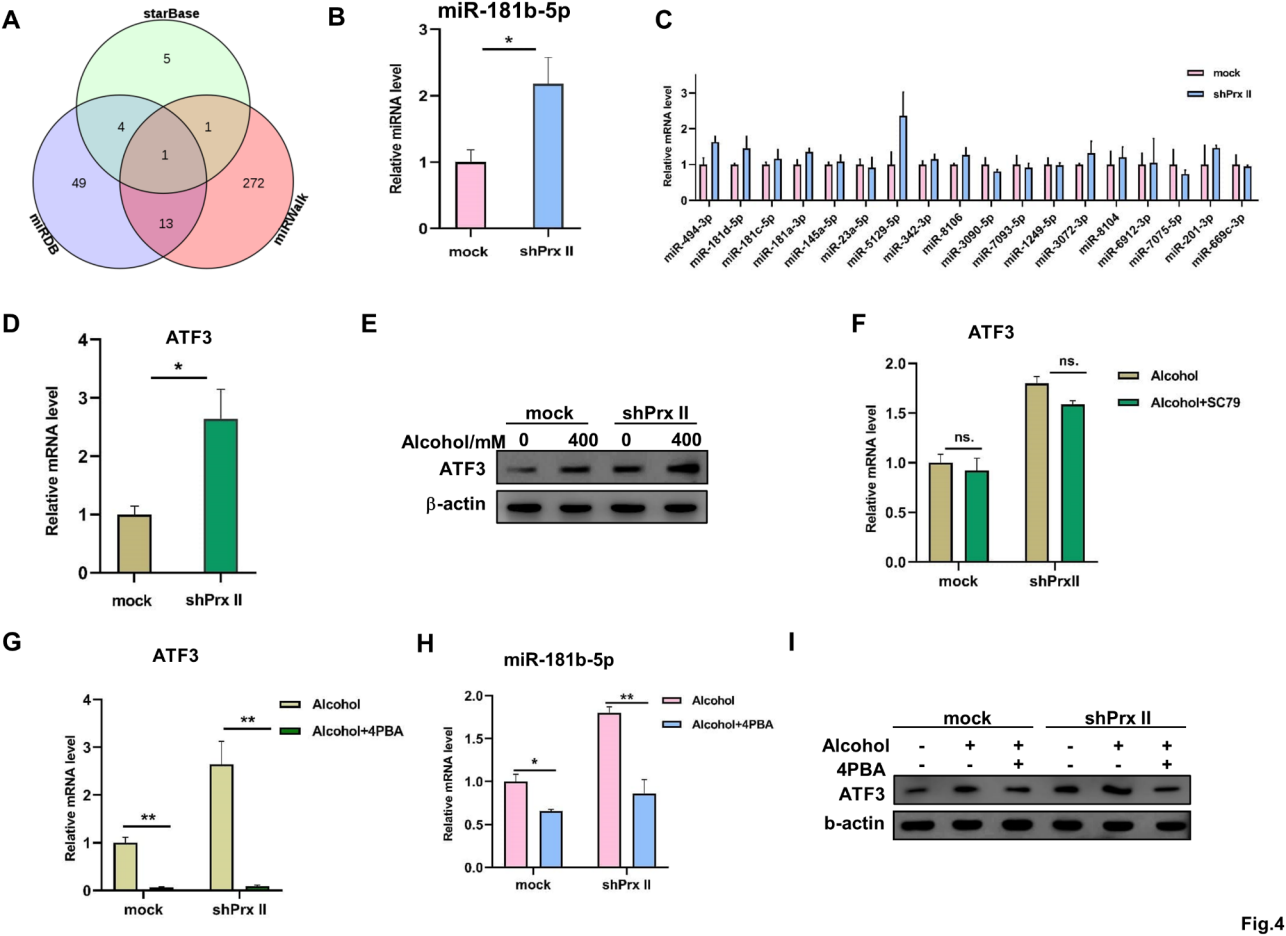
Prx II regulates Armcx3 expression by miR-181b-5p, through the transcription factor ATF3. (**A**) The miRWalk, miRDB, and starBase databases predicted the miRNAs that target Armcx3, which were plotted in a Venn diagram with reciprocal intersection. (**B, C**) The expression levels of miRNAs predicted by the miRWalk, miRDB, and starBase databases in mock HT22 and shPrx II HT22 cells following alcohol treatment, were detected using qRT-PCR. (**D**) ATF3 mRNA expression levels were detected using qRT-PCR. (**E**) Western blotting was used to detect ATF3 protein expression levels. (**F**) ATF3 mRNA expression after the addition of SC79 was detected using qRT-PCR. (**G**) ATF3 mRNA expression after the addition of 4PBA was detected using qRT-PCR. (**H**) The expression levels of miR-181b-5p after 4PBA pretreatment were detected using qRT-PCR. (**I**) Western blotting was used to detect Armcx3 protein expression levels after 4PBA pretreatment. Each analysis was performed in triplicate

To investigate the potential miRNA-mediated regulation of Armcx3 by Prx II, we utilized bioinformatics tools miRWalk [14], miRDB [15], and starBase [16] to predict candidate miRNAs targeting Armcx3. This analysis yielded 18 intersecting miRNA candidates (Fig. 4A; Table 1). In shPrx II HT22 cells post-alcohol treatment, miR-181b-5p was upregulated, while the expression of other miRNAs remained unchanged (Fig. 4B and C). Using the NCBI and JASPAR database predictions, we identified ATF3 binding sites in the promoter regions of miR-181b-1 and miR-181b-2 (Tables 3 and 4). Protein and RNA expression analyses revealed that ATF3 levels were upregulated in shPrx II HT22 cells, compared to controls, post-alcohol treatment (Fig. 4d, e; Fig. S5A). To discern the pathway through which ATF3 is regulated, HT22 cells were pretreated with the AKT activator SC79 [20] and the ERS inhibitor 4PBA [21], before alcohol exposure. While SC79 had no significant effect, 4PBA pretreatment attenuated the alcohol-induced upregulation of ATF3 (Fig. 4F and G) and the downregulation of miR-181b-5p (Fig. 4H). Additionally, 4PBA prevented the increase in ATF3 expression levels (Fig. 4I and S5B). These findings suggested that Prx II modulates Armcx3 expression through an ATF3-miR-181b-5p axis, in response to alcohol-induced stress.

**Table 3:** Transcription factor binding sites

2 putative sites were predicted with these settings (85%) in sequence named miR-181b-1							
Model ID	Model name	Score	Relative score	Start	End	Strand	predicted site sequence
MA1988.1	ATF3	8.365428	0.8685369295284081	1223	1233	-	TATGACACAAG
MA1988.1	ATF3	7.5087614	0.8508471818109317	1628	1638	+	TTTGACACAGC

**Table 4:** transcription factor binding sites

7 putative sites were predicted with these settings (85%) in sequence named miR-181b-2							
Model ID	Model name	Score	Relative score	Start	End	Strand	predicted site sequence
MA0605.1	ATF3	8.1040325	0.8776100086375695	1902	1909	-	GATGACTA
MA0605.1	ATF3	7.9220777	0.8738409151027318	354	361	+	TATGACTT
MA0480.1	ATF3	9.048631	0.8971768338788214	188	195	-	GATGACTC
MA1988.1	ATF3	13.939148	0.983631534943882	185	195	-	GATGACTCAGC
MA1988.1	ATF3	10.228355	0.9070054746101427	185	195	**+**	GCTGAGTCATC
MA1988.1	ATF3	10.020256	0.9027083239778954	1225	1235	-	AATGACACATT
MA1988.1	ATF3	9.709189	0.8962849494830348	1377	1387	+	AGTTACTCACT

### Reintroduction of Prx II restores mitochondrial function in HT22 cells

**Fig. 5:**
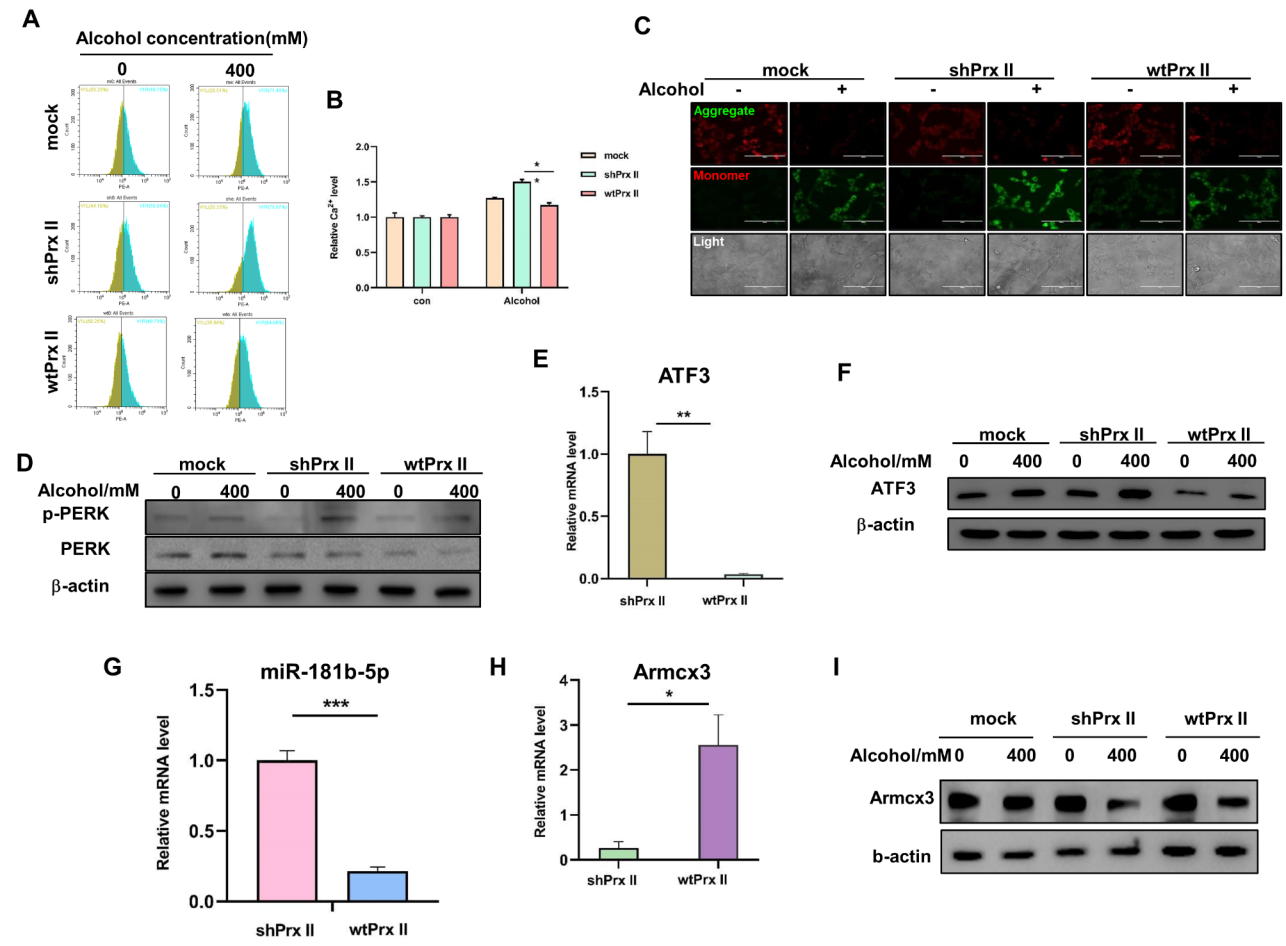
Re-transfer of Prx II gene to identify the mechanism through which Prx II protects alcohol-induced decrease in mitochondrial membrane potential. (**A**) Flow cytometric detection of intra-mitochondrial Ca^2+^ levels. (**B**) Quantitative analysis of the data in panel “A.” (**C**) Fluorographic detection of mitochondrial membrane potential. (**D**) Western blotting was used to detect PERK signaling pathway protein expression levels. (**E**) ATF3 mRNA expression levels were detected using qRT-PCR. (**F**) Western blotting was used to detect ATF3 protein expression levels. (**G**) The expression levels of miR-181b-5p were detected using qRT-PCR. (**H**) Armcx3 mRNA expression levels were detected using qRT-PCR. (**I**) Western blotting was used to detect Armcx3 protein expression levels. Each analysis was performed in triplicate

The reintroduction of Prx II into shPrx II HT22 cells (wtPrx II HT22 cells) led to a significant increase in Prx II protein levels and restoration of mitochondrial function after alcohol treatment, as evidenced by normalized Ca^2+^ levels, mitochondrial membrane potential, and the downregulation of PERK signaling and ATF3 expression (Fig. 5A–I; FIG. S6). These results confirmed the protective role of Prx II in regulating mitochondrial membrane potential via ERS and associated signaling pathways.

### Prx II attenuates alcohol-induced ERS by scavenging ROS

**Fig. 6:**
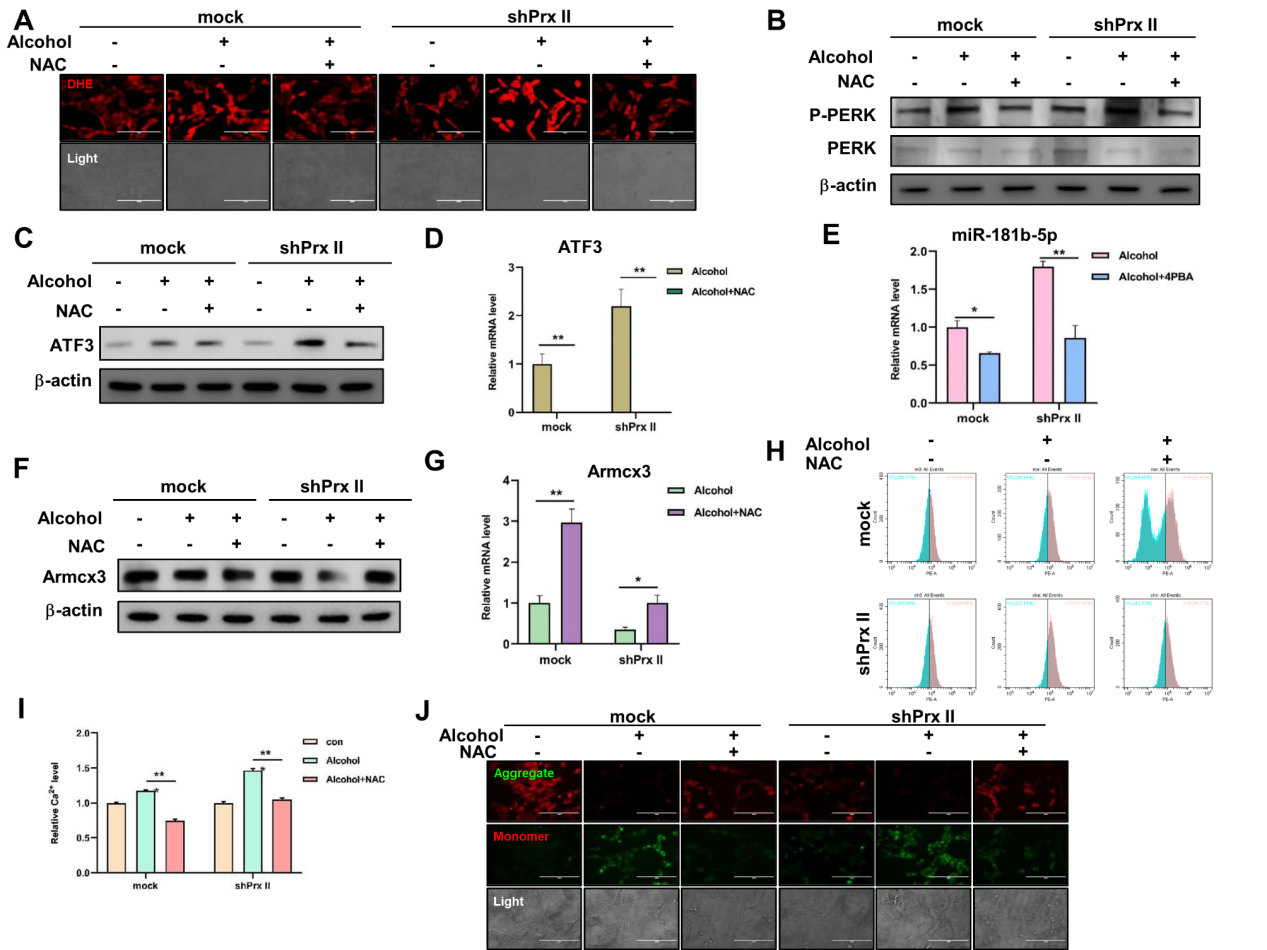
Prx II protects alcohol-induced decrease in mitochondrial membrane potential by scavenging ROS. (**A**) Detection of intracellular ROS levels in HT22 cells after alcohol treatment using fluorography. (**B**) Detection of the PERK signaling pathway after NAC pretreatment using western blotting. (**C**) Detection of ATF3 protein expression levels after NAC pretreatment using western blotting. (**D**) Detection of ATF3 mRNA expression levels after NAC pretreatment using qRT-PCR. (**E**) Detection of miR-181b-5p expression level after NAC pretreatment using qRT-PCR. (**F**) Detection of Armcx3 protein expression level after NAC pretreatment using western blotting. (**G**) Detection of Armcx3 mRNA expression level after NAC pretreatment using qRT-PCR. (**H**) Detection of Ca^2+^ level in mitochondria after NAC pretreatment using flow cytometry. (**I**) Quantitative analysis of the data in panel “h.” (**J**) Detection of mitochondrial membrane potential after NAC pretreatment using fluorography. Each analysis was performed in triplicate

Prx II confers protection against mitochondrial damage by scavenging intracellular ROS, which potentially mitigates ERS induced by alcohol metabolism. We observed that ROS levels were elevated in Prx II-deficient cells post-alcohol treatment, which was reversed by the ROS scavenger NAC. This led to the inhibition of the PERK signaling pathway, downregulation of ATF3 and miR-181b-5p expression, upregulation of Armcx3, and normalization of intra-mitochondrial Ca^2+^ levels and mitochondrial membrane potential (Fig. 6A–J; Fig. S7). These results suggested that Prx II protects against mitochondrial damage by counteracting alcohol-induced ERS through ROS scavenging.

## Discussion

In this study, we explored the role of Prx II in protecting the mitochondria from oxidative stress-induced damage, focusing on elucidating the precise molecular mechanisms involved. Our investigation revealed that Prx II is instrumental in regulating EMCSs by fostering the transcription of ATF3, which in turn activates miR-181b-5p expression. This sequence of events leads to the suppression of Armcx3, a gene implicated in mitochondrial aggregation, and is facilitated by the PERK signaling pathway. These processes facilitate the preservation of mitochondrial integrity under oxidative stress conditions.

Utilizing transmission electron microscopy to examine alcohol-treated HT22 cells, we observed a marked elongation of mitochondria-associated ER membranes as well as an increase in the percentage of bodies in contact with mitochondria in Prx II-depleted cells. The intimate proximity of the ER to mitochondria, typically within 10–30 nm [22], highlights the significance of EMCSs in the regulation of mitochondrial function, acting as pivotal conduits for Ca^2+^ transfer, thereby influencing mitochondrial membrane potential [10–12]. Our analysis of mitochondrial Ca^2+^ levels in alcohol-exposed HT22 cells revealed a pronounced increase in Prx II-deficient cells, which was reversed by the Ca^2+^ chelator BAPTA, restoring mitochondrial membrane potential and levels of proteins associated with mitochondria-dependent apoptosis. These findings underscore the regulatory role of EMCSs in mitigating mitochondrial damage and apoptosis, with Prx II playing a pivotal role in this regulatory process.

The distribution of mitochondria within cells is a key determinant of EMCS formation [12], and disruption of the intracellular mitochondrial transport leads to their accumulation around the nucleus and enhances contact with the ER, promoting EMCS formation [23, 24]. In shPrx II HT22 cells treated with alcohol, there was a significant aggregation of mitochondria around the nucleus, compared to control cells. RNA sequencing analysis of alcohol-treated mock and Prx II-depleted HT22 cells highlighted three differentially expressed genes associated with mitochondrial localization: Armcx3, Rhot2, and Cluh. Notably, Armcx3, which is localized in the mitochondria in neuronal cells and is involved in mitochondrial transport through its interaction with Miro and Trak2 [18], was downregulated in Prx II-depleted cells. We also investigated miRNAs, which regulate mRNA expression by binding with complementary sequences and causing mRNA degradation or translation inhibition [25], as potential mediators of the effects of Prx II on Armcx3. Using a series of databases (miRTarBase, miRDB, and miRWalk) and real-time quantitative reverse transcription PCR (qRT-PCR) screenings, we revealed that miR-181b-5p was upregulated in Prx II-depleted cells, implicating it in the regulation of Armcx3 expression.

Transcription factors are known to modulate miRNA expression by binding to promoter regions upstream of miRNA genes [26] and proteins of the Prx family regulate miRNA expression through transcription factors [26]. Utilizing the JASPAR database, we predicted that ATF3 can bind to the upstream promoter regions of the miR-181b-5p precursors, miR-181b-1 and miR-181b-2. ATF3, a stress-responsive transcription factor, is typically expressed at extremely low levels unless the cells are stimulated [27–29]. We confirmed the expression of ATF3 at both the RNA and protein levels following cellular stress. Interestingly, while the PI3K-AKT signaling pathway can regulate ATF3 expression via S6K activation [30, 31], our findings suggest that the PERK signaling pathway, a component of the unfolded protein response to ERS [32], is the predominant pathway through which Prx II affects ATF3 and miR-181b-5p expression.

The PERK signaling pathway is part of the unfolded protein response downstream of ERS [33]. Previous research has shown that Prx IV, located in the ER, directly influences ERS [34]. However, the role of Prx II, predominantly found in the cytoplasmic matrix [35], in the regulation of ERS was unclear. Our observations of significant ER swelling in Prx II-depleted HT22 cells, along with the inhibition of alcohol-induced PERK signaling by the ERS inhibitor 4PBA, provide new insights into the regulatory functions of Prx II. These findings suggest that Prx II may play a crucial role in preventing ERS and subsequent mitochondrial damage by detoxifying ROS generated during alcohol metabolism.

The unique structural features of the proteins of the Prx family, including conserved cysteine residues, enable them to effectively scavenge ROS [36]. The differential cellular distribution of Prx family members, with Prx III in mitochondria [37], Prx II in the cytoplasmic matrix [38], and Prx IV in the ER [34], was previously thought to dictate their specific organelle-related functions. However, our study has demonstrated that Prx II can also influence organelle interactions and physiological functions beyond its cytoplasmic matrix localization, thus expanding the scope of functional studies within the Prx family. Despite these advancements, the potential functional similarities between Prx II and Prx I, both cytoplasmically located and containing two conserved cysteine residues [38], are yet to be elucidated. Future research should clarify the distinct and possibly overlapping roles of these peroxiredoxins, particularly in the context of oxidative stress-induced mitochondrial membrane potential decline.

## Conclusions

**Fig. 7:**
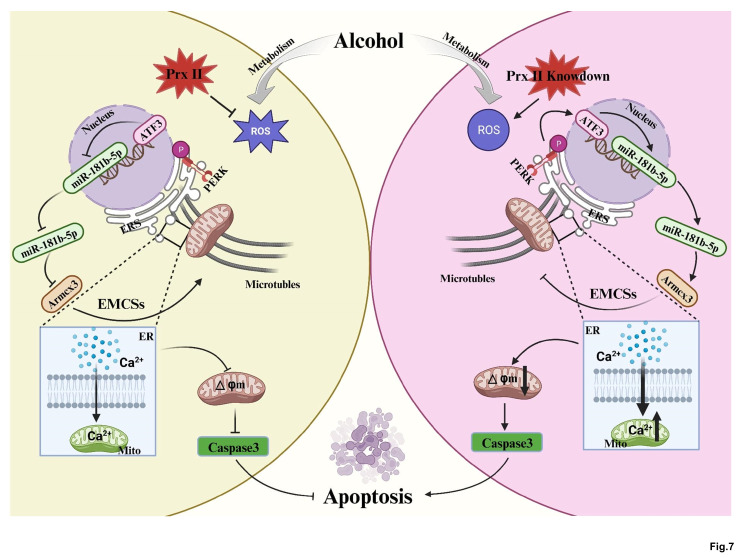
Prx II is a promising target for the treatment of neurodegenerative diseases induced by oxidative stress via mitochondrial membrane potential decrease. By regulating EMCSs, Prx II can prevent the accumulation of Ca^2+^ in the mitochondrial matrix, which can stimulate the formation and opening of the MPTP, leading to the release of Ca^2+^ and apoptotic factors into the cytoplasm, and the induction of cell death

In conclusion, our research offers novel insights into the protective role of Prx II in mitigating oxidative stress-induced mitochondrial damage, and provides a foundation for further exploration into its therapeutic potential against neurodegenerative diseases driven by oxidative stress and mitochondrial dysfunction (Fig. 7). The interplay between Prx II, ATF3, and miR-181b-5p, with its implications for mitochondrial transport and EMCS formation, represents a promising target for novel therapeutic interventions.

### Electronic supplementary material

Below is the link to the electronic supplementary material.

Supplementary Material 1

## Data Availability

All data that support the findings of this study are available from the corresponding authors upon reasonable request.

## Abbreviations

Prx II: Peroxiredoxin II
ROS: Reactive oxygen species
ER: Endoplasmic reticulum
ERS: Endoplasmic reticulum stress
EMCSs: ER-mitochondrial contact sites
GO: Gene ontology
