# Supplementary material for: Exploring the role of Prx II in mitigating endoplasmic reticulum stress and mitochondrial dysfunction in neurodegeneration
Source: Cell Commun Signal. 2024 Apr 18;22:231. doi: 10.1186/s12964-024-01613-x (PMC11025193; doi:10.1186/s12964-024-01613-x)
Supplement: Supplementary file 1 — Supplementary Material 1 [file 12964_2024_1613_MOESM1_ESM.docx]

**Supplementary Figures**

**
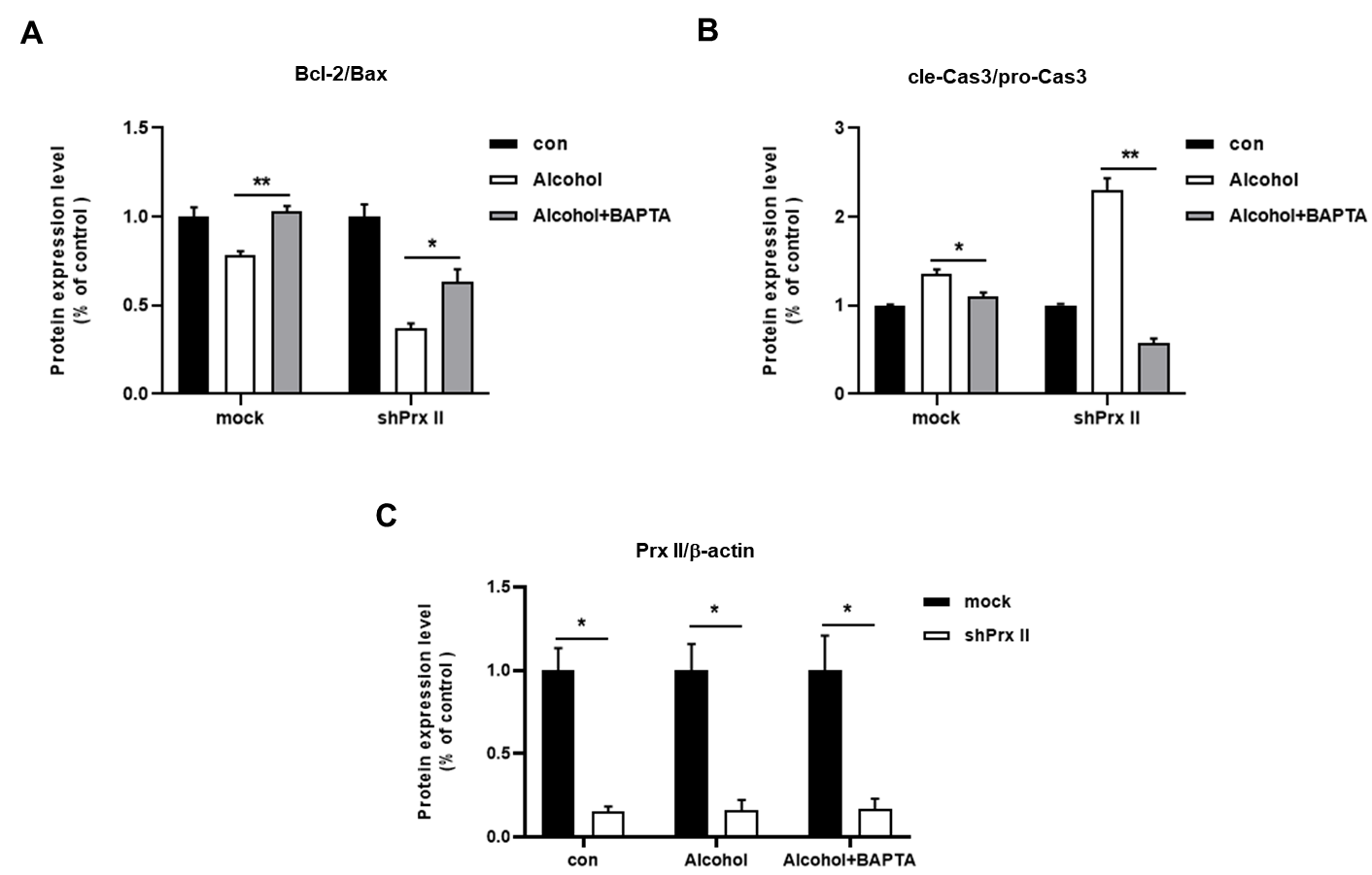
**

**Supplementary Figure. 1. (A, B)** Quantitation of western blotting detection of mitochondria-dependent apoptosis-related protein expression levels after BAPTA pretreatment; **(C)** quantitation of western blotting to detect Prx II protein expression levels after BAPTA pretreatment.

**
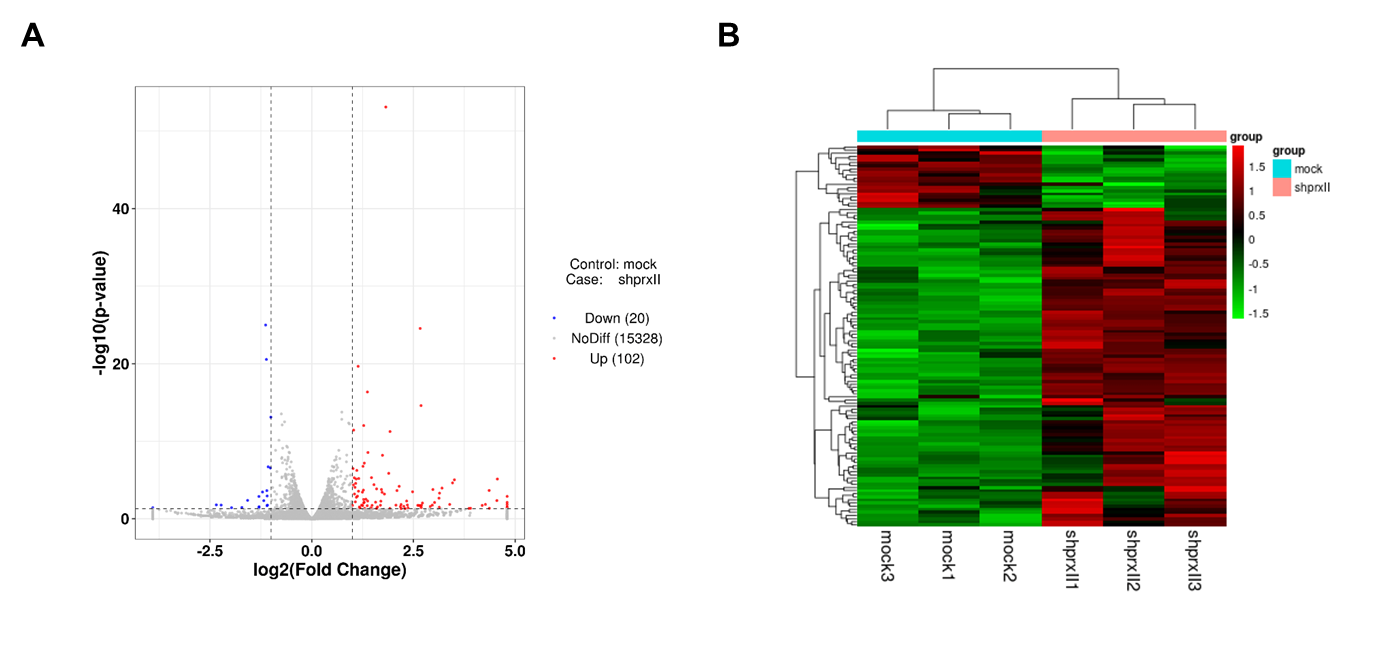
**

**Supplementary Figure. 2. (A)** mock and shPrx II alcohol-treated RNA sequencing volcano maps, with red for up-regulated genes and blue for down-regulated genes；**(B)** Differential gene heat map.

**
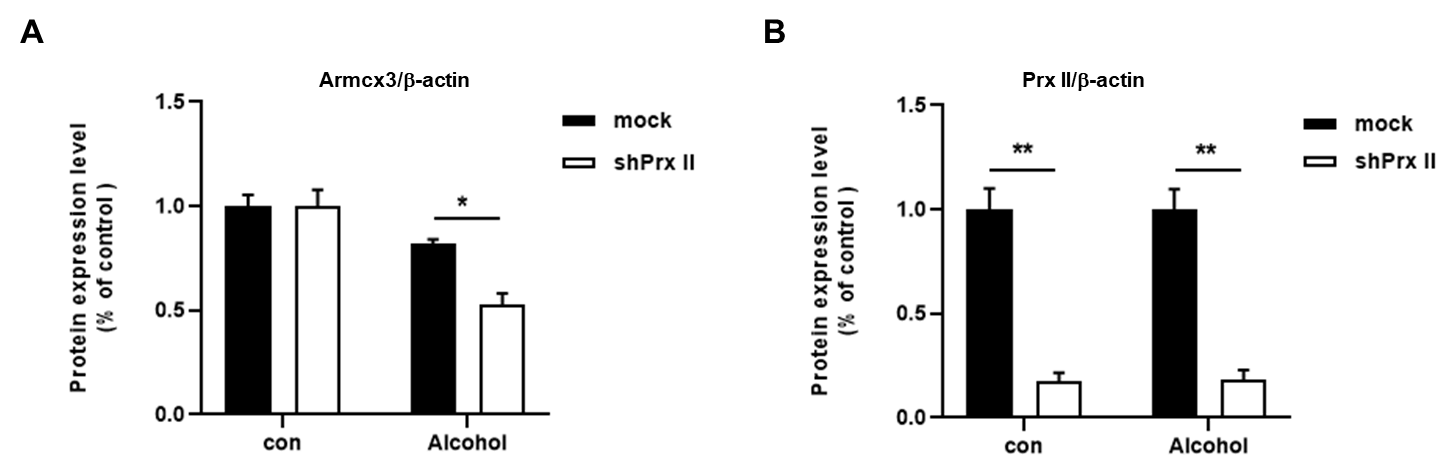
**

**Supplementary Figure. 3. (A)** Quantitation of western blotting to detect Armcx3 protein expression levels after alcohol treatment; **(B)** Quantitation of western blotting to detect Prx II protein expression levels after alcohol treatment.

**
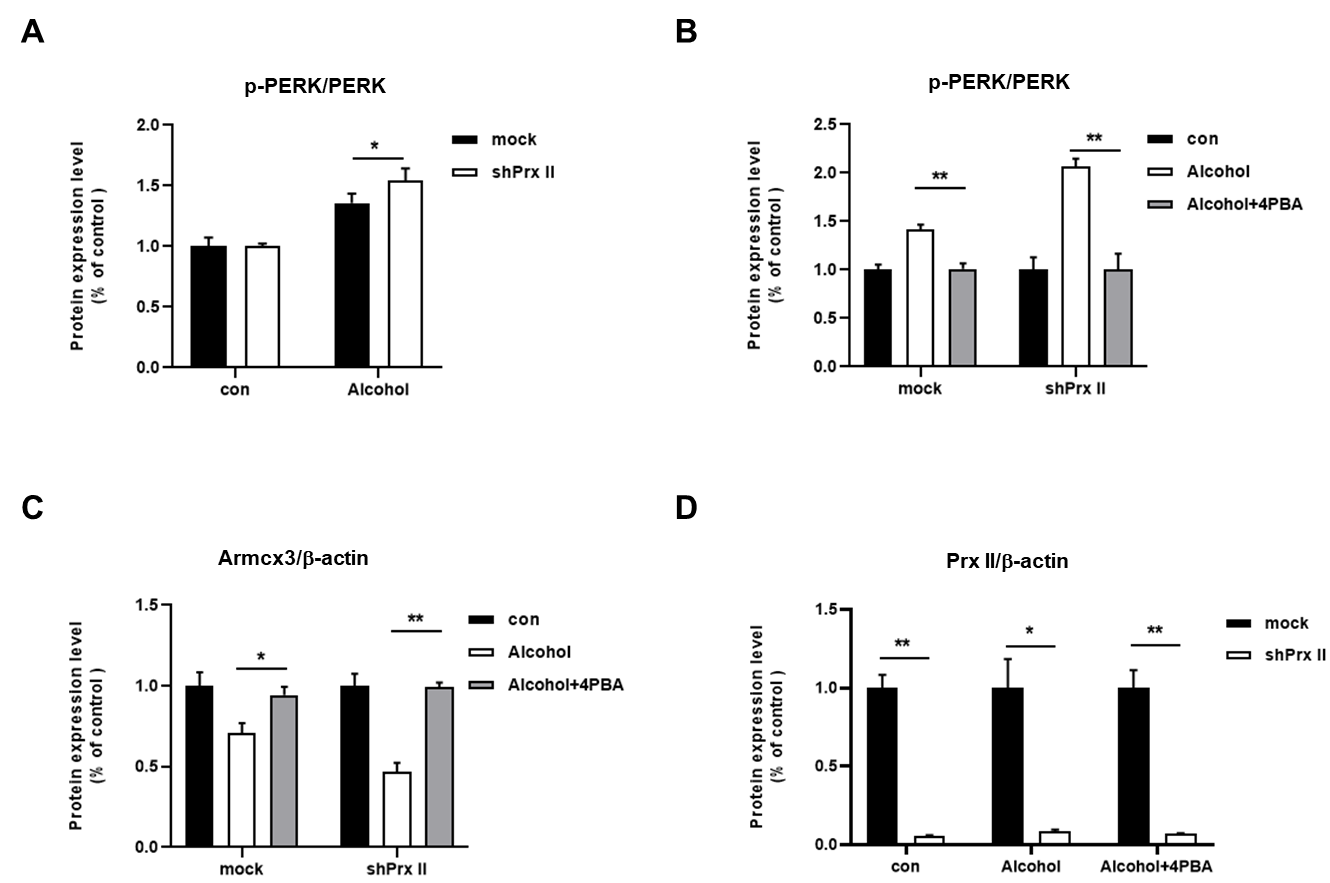
**

**Supplementary Figure. 4. (A)** Quantitation of western blotting to detect p-PERK protein expression levels after alcohol treatment; **(B)** Quantitation of western blotting to detect p-PERK protein expression levels after 4PBA pretreatment; **(C)** Quantitation of western blotting to detect Armcx3 protein expression levels after 4PBA pretreatment; **(D)** Quantitation of western blotting to detect Prx II protein expression levels after 4PBA pretreatment.

**
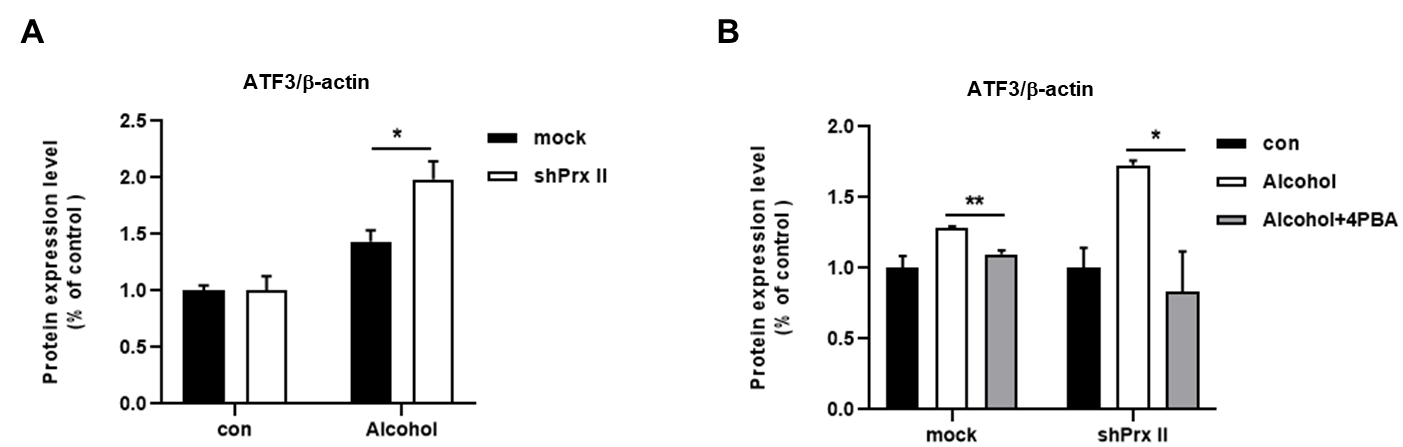
**

**Supplementary Figure. 5. (A)** Quantitation of western blotting to detect ATF3 protein expression levels after alcohol treatment; **(B)** Quantitation of western blotting to detect ATF3 protein expression levels after 4PBA pretreatment.

**
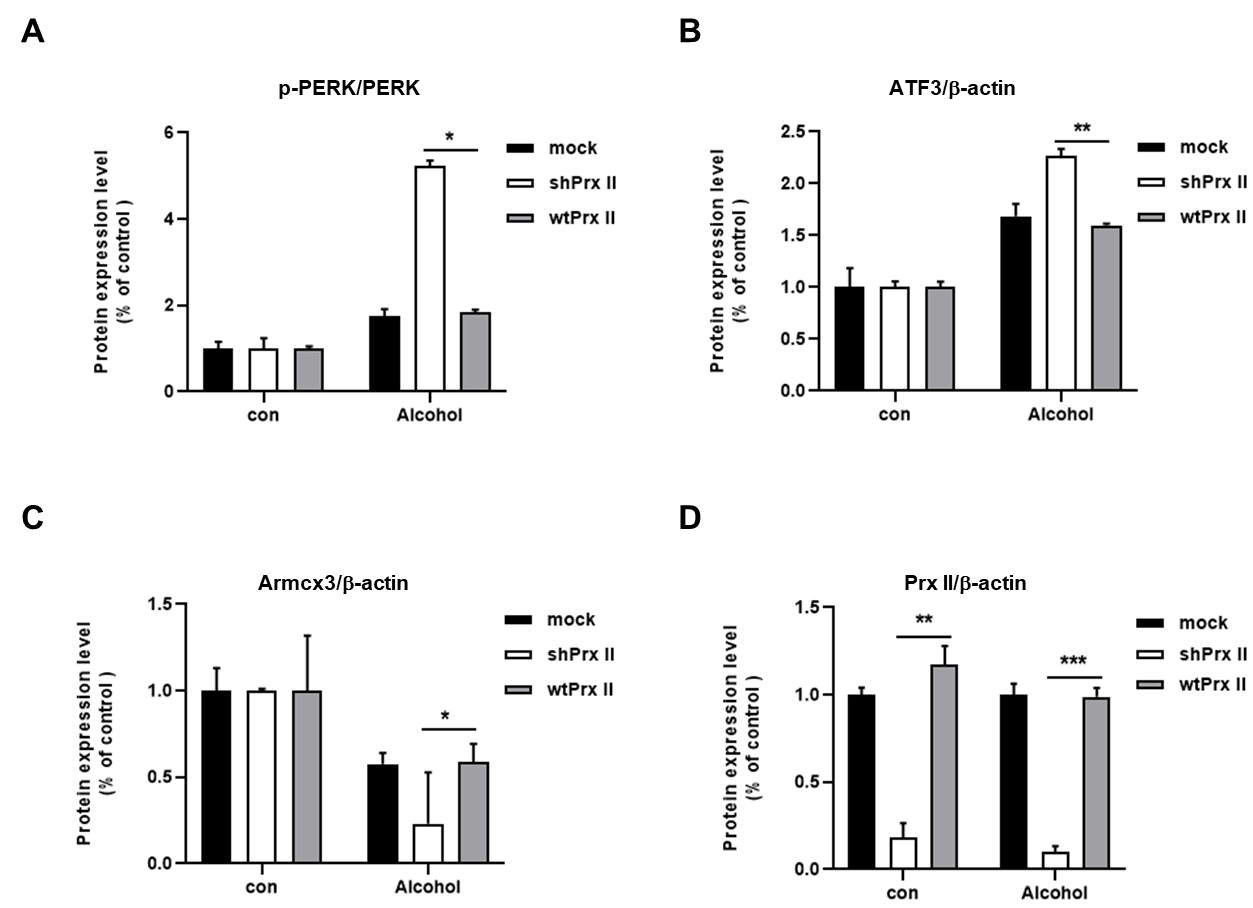
**

**Supplementary Figure. 6. (A)** Quantitation of western blotting to detect p-PERK protein expression levels in wtPrx II HT22 cells; **(B)** Quantitation of western blotting to detect ATF3 protein expression levels in wtPrx II HT22 cells; **(C)** Quantitation of western blotting to detect Armcx3 protein expression levels in wtPrx II HT22 cells; **(D)** Quantitation of western blotting to detect Prx II protein expression levels in wtPrx II HT22 cells.


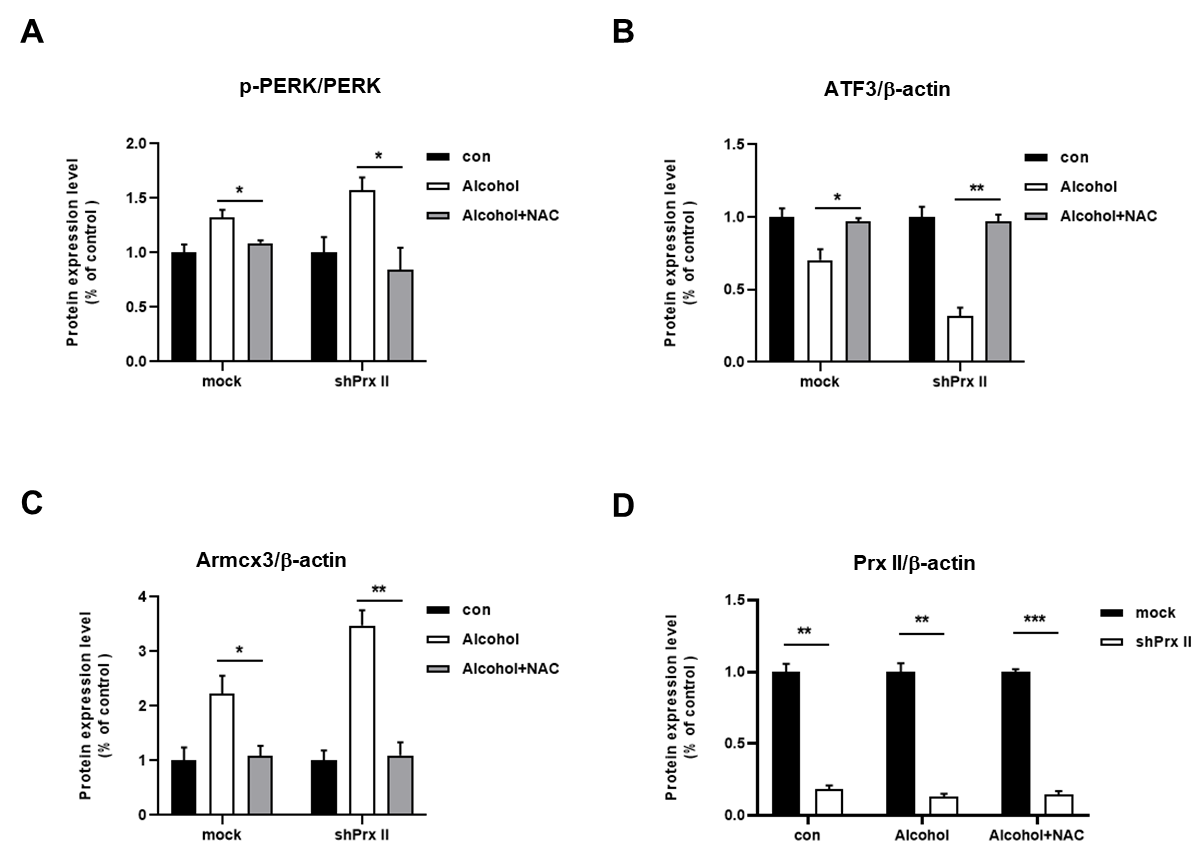


**Supplementary Figure. 7. (A)** Quantitation of western blotting to detect p-PERK protein expression levels after NAC pretreatment; **(B)** Quantitation of western blotting to detect ATF3 protein expression levels after NAC pretreatment; **(C)** Quantitation of western blotting to detect Armcx3 protein expression levels after NAC pretreatment; **(D)** Quantitation of western blotting to detect Prx II protein expression levels after NAC pretreatment.
